# Worker knowledge of occupational legislation and related health and safety benefits

**DOI:** 10.4102/curationis.v41i1.1869

**Published:** 2018-06-28

**Authors:** Mogale L. Pilusa, Mataniele S. Mogotlane

**Affiliations:** 1 Department of Nursing Science, Tshwane University of Technology, South Africa

## Abstract

**Background:**

Environmental hazards such as fumes, gases, poor ventilation and extreme temperatures might lead to occupational illnesses and injuries. To protect workers from potential occupational health and safety risks, the government has promulgated occupational legislation that must be implemented in work settings.

**Objectives:**

The objective of this study was to explore the knowledge of workers about occupational legislation and its benefits for their health and safety. The aim was to make recommendations that would be implemented to improve the knowledge and compliance with occupational legislation as advised by the occupational health nurse.

**Methods:**

A quantitative approach was used for this study. One hundred and ten respondents were sampled from an accessible population of 1590 workers. A self-administered questionnaire was used for gathering data. Data were analysed through descriptive analysis using Statistical Package for Social Sciences.

**Results:**

Research findings indicated that only 18 (16.36%) respondents were knowledgeable about occupational legislation. Eighty-two (75.54%) respondents were unable to mention even one occupational act by name. Respondents mentioned five benefits of occupational legislation for the health and safety of workers. These benefits were that legislation ensured safety, gave rights to workers, ensured compensation for occupational injuries and illnesses, and provided guidance in the prevention of occupational injuries and illnesses.

**Conclusion:**

Most respondents (75.54%) were found not to be knowledgeable about occupational legislation. The positive finding was that respondents knew the benefits of occupational legislation. To improve the respondents’ knowledge about legislation, five recommendations are made by the researcher.

## Introduction

Even though work and work settings such as manufacturing and mining industries are an important part of our lives, they may be a source of ill health among workers because of unsafe working environments and work processes (Bansah, Yalley & Dumakor-Duey 2016:8). Workers might be exposed to toxic chemicals in the workplace. In addition, they might be exposed to excessive noise, extreme temperatures and dust, as well as poor ventilation (Bansah et al. 2016:14). These occupational health and safety risks can lead to occupational injuries and illnesses. According to Hattingh and Acutt (2016:189) one of the roles of the occupational health nurse is to advise management and workers about occupational legislative requirements to ensure legal compliance at the workplace. The occupational health nurse’s responsibility is to secure the health, safety and well-being of the workforce (Michell 2011:37).

### Problem statement

In their research, Sieberhagen, Rothmann and Pienaar (2009:1) reported that globally there are 270 million occupational accidents, 160 million occupational illnesses and two million fatalities. For workers to be protected against occupational health and safety risks, the South African government has promulgated occupational health legislation with the purpose of ensuring safety at work. Proper implementation of occupational legislation enhances safe work processes and working environments. The challenge with the implementation of occupational legislation is that workers’ knowledge of occupational legislation is limited and adherence is thus impaired.

### Background

According to the *Occupational Health and Safety Act* (South Africa 1993b:section 8), employers have to provide and maintain a work environment that is safe for employees. This is done to minimise all health and safety risks that can affect employees. Employees also have a duty to take care of their health and safety, comply with all lawful orders and report unhealthy and unsafe situations and incidents that may affect their health or cause them injuries (South Africa 1993b:section 14). It is beneficial for employees to know occupational legislation so that they are able to comply with it and be able to inform employers if their workplaces are unsafe.

Despite occupational legislation being in place, there are many reported health and safety incidents, including both occupational injuries and illnesses. It is reported that worldwide, every 15 seconds, there is a fatality and 153 workers sustain occupational injuries in the same time span (Hattingh & Acutt 2016:65). Mining Safety (2012:1) indicates that there were 168 deaths in South African mines in 2009, 128 in 2010 and 116 in 2011. These deaths were reportedly caused by occupational hazards in work settings and a push by management for higher production targets, regardless of the safety standards in place. Furthermore workers are also exposed to occupational diseases such as silicosis and tuberculosis (TB). The TB mortality rate in the mines is higher than that of mine accidents (Murray, Davies & Rees 2011:67).

According to Hattingh and Acutt (2016:31) and Bansah et al. (2016:14), workers can be exposed to toxic chemical fumes, vapours, excessive dust, high noise levels, poor ventilation, poor illumination and harsh temperatures (too hot or too cold). These pose occupational safety risks at the workplace that can lead to occupational injuries, illnesses and, at worst, premature fatalities.

Some employees are forced to work non-stop in an unsafe work area for 9 hours a day, 6 days a week, with only a 15-min lunch break in the day (Piloso 2014:13). These extended hours expose employees to occupational health and safety risks. Chen and Xie (2014:87) emphasise the fact that rest periods and leave periods reduce contact with the work situation and the subsequent exposure to chemicals. Breaks away from work also substantially reduce fatigue-related injury risks. It has been noted that occupational legislation is beneficial to workers as it enforces aspects such as biological monitoring, medical surveillance and occupational hygiene (South Africa 1996:sections 12[1], 13[1]).

### Research objectives and aim

The objectives of this study were to:

- explore the knowledge of workers about occupational legislation
- determine awareness of respondents about the benefits of occupational legislation for workers in work settings.

The aim of the study was to make recommendations that would improve workers’ knowledge of occupational legislation.

## Research methods

### Design

A quantitative method using an exploratory, descriptive and contextual research design was used in four work settings in Phalaborwa, Limpopo Province of South Africa. In this study, an exploratory design was used to explore employees’ knowledge about occupational legislation and the benefits thereof, while a descriptive design was used to discover new meaning, namely, respondents’ knowledge of occupational legislation and the benefits thereof.

### Context of the study

The study was conducted at four conveniently selected work settings in Phalaborwa, Limpopo Province of South Africa. The four work settings comprised a mine, a company for construction work, a security and fencing company, and a company that provided drivers for mines around Phalaborwa.

### Population sample and sampling method

In this study, the target population was all workers regardless of trade. The accessible population of the study was workers in the selected work settings from which a sample was selected. The accessible population consisted of 1590 workers employed at the four selected work settings that took part in the study, from which 110 respondents were conveniently and purposively sampled using a non-probability sampling technique.

### Data collection and analysis

A self-administered questionnaire with both closed- and open-ended questions was used to gather data. Hard copies of the questionnaire were distributed with the assistance of two field workers to those workers who had signed a consent form and were willing to participate in the study following an explanation on what the study entailed. The questionnaires were returned to the researcher after completion. Data analysis was done by means of descriptive analysis using Statistical Package for Social Sciences.

### Validity

Validity of the data collection instrument was ensured by conducting a pretest. Content validity was ensured by including a variety of open-ended questions. Questions were formulated in simple, clear language and clear instructions were given to respondents for the completion of the questionnaire.

### Ethical considerations

Before commencement of the study, permission was obtained from the management of the four work settings that took part in the study. All respondents who took part in the study gave informed consent before taking part in the study. The study was approved by Tshwane University of Technology’s ethical research committee. The reference number for the ethics permit is Ref: REC2012/05/005(3).

## Results

### Demographic profile of respondents

The demographic profile for the study comprised of respondents’ educational level and employment duration in years.

### Respondents’ educational level

Fifty (45.45%) of the 110 respondents who took part in the study had worked for 0–5 years, 25 (22.72%) for 6–10 years, 8 (7.27%) for 11–15 years, 15 (13.63%) for 16–20 years and 12 (10.90%) had worked for more than 20 years. The implication of work duration is that workers who have been working for 5 years or less might not be experienced enough at their work. Additionally, it is possible that they could have not received enough training on occupational legislation from their employers or managers.

### Formal education of respondents

Eight (7.27%) respondents had no formal education, 33 (30.00%) had only primary education, 26 (23.63%) had secondary education and 43 (39.09%) had tertiary education. The implication of no formal education and primary education among respondents is that they might encounter challenges in understanding occupational legislation. There might also be challenges with language where occupational legislation is introduced and written in English.

### Knowledge of occupational legislation

**FIGURE 1:**
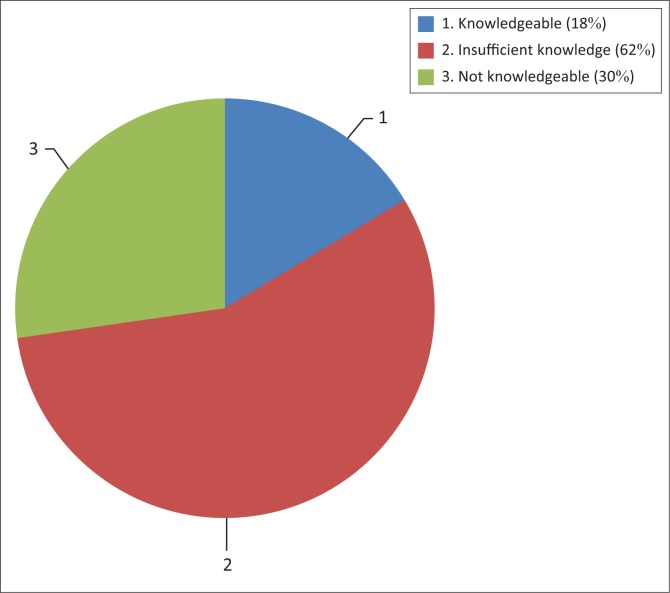
Participants’ knowledge of occupational legislation (*n* = 110).

The results on knowledge of occupational legislation are depicted in Figure 1. From the 110 respondents who took part in the study, only 18 (16.36%) indicated that that they were knowledgeable about occupational legislation. Sixty-two (56.36%) respondents reported having insufficient knowledge while 30 (27.27%) indicated that they were not knowledgeable about occupational legislation at all.

### Regular occupational legislation teaching sessions

Respondents were also asked whether there were regular sessions for teaching of occupational legislation at their work settings, and most of them (92; 83.64%) indicated that there were no regular sessions for teaching of occupational legislation at their work settings. Only 18 (16.36%) respondents indicated that there were regular sessions for teaching of occupational legislation at their workplace.

### Known occupational legislation

Respondents were given five occupational acts that were relevant to the study and asked to indicate those that they knew from the list. These five acts were the *Basic Conditions of Employment Act* (75/1997), the *Compensation of Occupational Injuries and Diseases Act* (130/1993), the *Occupational Health and Safety Act* (85/1993), the *Mine Health and Safety Act* (29/1996) and the *Employment Equity Act* (55/1998). As depicted in Table 1, 82 (74.54%) of the 110 respondents who took part in the study did not know a single occupational act, while the most known legislative act was the *Basic Conditions of Employment Act* (75/1997), which was known by 15 (13.63%) respondents. The least known legislative act was the *Employment Equity Act* (55/1998), which was known by six (5.45%) respondents. Some respondents mentioned more than one act.

**TABLE 1:** Participants’ knowledge of occupational legislative acts (*n* = 110).

Occupational legislation	Number	Percentage (%)
*Basic Conditions of Employment Act* (75/1997)	15	13.63
*Compensation of Occupational Injuries and Diseases Act* (130/1993)	8	7.27
*Occupational Health and Safety Act* (85/1993)	10	9.09
*Mine Health and Safety Act* (29/1996)	11	10.00
*Employment Equity Act* (55/1998)	6	5.45
None	82	74.54

### Health and safety benefits of occupational legislation

Regarding the health and safety benefits of occupational legislation for workers, five benefits were mentioned by respondents as indicated in Table 2. Thirty-eight respondents (34.54%) indicated that occupational legislation provided guidance in the prevention of occupation injuries and illnesses, 19 (17.27%) mentioned that it gives rights to employees such as a right to refuse to work under unsafe conditions, and 20 (18.18%) indicated that it ensured safety at the workplace. Ten respondents (9.09%) indicated that occupational legislation ensured compensation of workers for occupational injuries and illnesses and seven (6.36%) indicated that it prevented exploitation of employees by employers. Some respondents indicated more than one benefit, while 27 (24.54%) did not know a single benefit of occupational legislation.

**TABLE 2:** Health and safety benefits of occupational legislation for workers as indicated by respondents (*n* = 110).

Health and safety benefits of occupational legislation	Number	Percentage (**%**)
Guides in prevention of occupational injuries and illnesses	38	34.54
Ensures safety at work	20	18.18
Gives rights to employees	19	17.27
Ensures compensation of workers for illnesses and injuries	10	9.09
Prevents exploitation of employees	7	6.36
None	27	24.54

## Discussion

### Knowledge of occupational legislation

As depicted in Table 1, the results indicate that 16.36% of the respondents were knowledgeable about occupational legislation. Employees with insufficient knowledge and with no knowledge about occupational legislation were rated together as ‘not knowledgeable’ because of the fact that for occupational legislation to be properly implemented and complied with, workers needed to be fully knowledgeable about it. According to Garnica and Barriga (2018:1), lack of knowledge and few available resources result in non-compliance to occupational legislation in work settings. Lack of resources to protect the health and safety of workers is regarded as lack of knowledge by managers and/or reluctance of workers to use personal protective equipment (PPE). All these aspects are enforced by occupational legislation and, if occupational legislation is known and complied with in work settings, it will result in safe work settings and safe work processes that benefit workers by ensuring their health and safety at their places of work.

The poor knowledge of occupational legislation can be attributed to poor literacy levels by some of the respondents as the findings indicated that 8 respondents (7.27%) had no formal education while 33 (30.00%) had only primary school education. Occupational legislation is mostly written in English and most teaching is done in English; therefore, respondents’ limited education levels may have contributed to lack of knowledge about occupational legislation. Reading legislative acts is not easy, as the language reads differently from regular English; hence it is difficult to those with low literacy to comprehend occupational legislation.

The other factor that contributed to lack of knowledge about occupational legislation in these work settings was lack of regular training sessions at workplaces, as reported by 92 respondents (83.63%). The employer has a duty to inform, train and supervise employees about health and safety at work through the teaching of occupational legislation, among others (South Africa 1993b:section 2[e]). Bahn and Barratt-Pugh (2014:148–149) emphasise that employers have an obligation to teach employees about safety culture, which can reduce occupational injuries and illnesses. Teachings about safety culture and reduction of occupational injuries and illnesses are well documented in occupational legislation.

Employers have the duty to inform workers about work-related hazards for their health and safety (South Africa 1993b:section 13[a]). Workers’ knowledge of occupational legislation has to be increased and reinforced by providing information, instruction and training to employees to enable them to work safely, in a safe environment (South Africa 1996:section 1[a]). According to Bahn and Barratt-Pugh (2014:148–149), training of workers by employers should ensure a culture of safety and help in reducing occupational injuries. The training and education of employees about workplace safety is emphasised by Hattingh and Acutt (2016:67). This training should include occupational legislation and equipment usage. The authors warn that workers who are not properly trained are a risk to themselves and fellow workers.

### Health and safety benefits of occupational legislation

As shown in Table 2, respondents in the study indicated five different health and safety benefits of occupational legislation. The benefits mentioned were that occupational legislation provides guidance in the prevention of occupational injuries and illnesses, affords workers the right to refuse to work in unsafe settings, as well as the right to free medical surveillance. Occupational legislation ensures safety at the workplace in that it compels safety training of employees and supervision on compliance in relation to PPE usage, facilitates compensation of workers for occupational injuries and diseases, and ensures prevention of exploitation by employers in line with other related legislation such as *Basic Conditions of Employment Act* (75/1997) and *Employment Equity Act* (55/1998).

In the prevention of occupational injuries and diseases in work settings, the *Occupational Health and Safety Act* (South Africa 1993b:section 8[1]) and the *Mine Health and Safety Act* (South Africa 1996:section 2) concur with research findings, as they both urge employers and mine owners to provide conditions of safety and healthy work environments. These two legislative acts also require employers to provide free, effective PPE to workers. Most work settings are still unsafe because of lack of compliance to occupational legislation and, according to Sieberhagen et al. (2009:1), legislation is necessary to ensure that workers’ safety and wellness are taken seriously by employers. The occupational health nurse has a duty to increase awareness of health risks that impact the workers’ health and safety, as well as the measures that can mitigate the risks (Michell 2011:48).

Leffakis and Schoff (2012:49) indicate that a lack of safety in work settings can impact negatively on the workers’ health, morale and performance and can also result in safety claims, increased workers’ compensation premiums, litigations and loss of experienced workers because of occupational injuries, illnesses and/or fatalities. For general safety promotion, safety newsletters can be utilised by employers to impart safety-related information to workers (Salazar 2006:282). Safety in the workplace is the responsibility of not only the employer but also the workers, as stipulated by Lemmer (2016:35), as workers have to carry out instructions from employers to ensure safety and use prescribed PPE when required. Workers have the responsibility and obligation to comply with all the workplace procedures and policies.

Nineteen respondents (17.27%) indicated that occupational legislation affords rights to workers in the workplace. Michell (2011:84) alludes to this finding by indicating that occupational legislation affords workers the following rights: the right to leave an unsafe workplace; the right to medical examination and tests, which the employer has to pay for; the right to an exit medical examination and the right to be granted an exit medical certificate. Occupational legislation also affords workers the right to rest periods, which help workers to rejuvenate and reduce fatigue-related injury risks (Chen & Xie 2014:87; Fritz, Ellis, Demsky, Lin & Guros 2013:274). These rest periods and breaks are vital for replenishment of energy. Hattingh and Acutt (2011:33) add that occupational legislation affords workers the right to freedom from discrimination based on gender, sexual orientation, HIV status or religious beliefs. Occupational legislation, according to Bezuidenhout, Garbers and Potgieter (2007:15), also affords workers the right to freedom of association, according to which they can join a trade union of their choice, the right to strike and the right to protection against unfair labour practices such as unfair dismissal.

According to 10 respondents (9.09%), workers benefit from occupational legislation as they are compensated for occupational injuries and illnesses. Hattingh and Acutt (2011:71) indicate that workers or their legal dependents must be compensated for an occupational injury, death or any occupational illness with which a worker is diagnosed. This compensation is for medical expenses and loss of income because of occupational injury, illness or fatality. To ensure safety in work settings, the commissioner can penalise employers with poor records for safety (South Africa 1993a:section 85[1]). Even though this is a good gesture to curb occupational injuries and illnesses, Rappin, Wuellner and Bonauto (2016:353) argue that it can result in under-reporting of cases by employers.

Another health and safety benefit of occupational legislation for workers is that it helps to prevent exploitation of workers by employers in work settings, as indicated by eight participants (6.50%) in this study. To this effect, it has been reported by Ioannides, Oxousi and Mavroudeas (2014:45) that contrary to the *Basic Conditions of Employment Act*, which stipulates that employers must pay workers for overtime worked, some workers have been forced to work overtime without being paid for it and threatened with dismissal if they refused to work for this unpaid overtime. The implication of this forced overtime is that it increases the risk for illnesses such as myocardial infarction and diabetes mellitus, as well as the risk for muscular and skeletal discomfort. Workers can also develop stress, fatigue and depression, which predispose them to occupational injuries, while others can experience inadequate sleep, restlessness, deficits in performance and poor reaction time, which can also lead to occupational injuries (Bae 2012:60).

### Recommendations to facilitate knowledge about occupational legislation

To facilitate knowledge about occupational legislation that would enhance the health and safety of workers at work settings, five recommendations were suggested. The recommendations were that in work settings the occupational health nurse must facilitate regular training sessions about occupational legislation. The training sessions must be conducted in a language that workers can understand. Management should support and capacitate the occupational health nurse to provide meaningful training sessions for both employers and workers about knowledge and application of occupational legislation. Consultants may also be engaged to address specific legislative issues related to occupations. Furthermore the occupational health nurse must ensure that work settings display hard copies of occupational legislation (written in different languages to accommodate employees), in compliance with legislative requirements. Work settings, with the input of the occupational nurse, must formulate policies derived from occupational legislation to facilitate health and safety as well as compensation to workers.

## Lessons learnt

Most workers are not knowledgeable about occupational legislation. Some workers do not know the benefits of occupational legislation.

## Conclusion

The study was conducted to determine the knowledge of workers about occupational legislation and its benefits. According to the findings, most of the respondents were not knowledgeable about occupational legislation, as only 16.36% knew about it. The lack of knowledge can be attributed mainly to the fact that there is a lack of training about occupational legislation in the workplaces, as reported by 83.63% of the respondents. Even with limited knowledge about occupational legislation, the respondents mentioned five benefits that are derived from the implementation of occupational legislation. Five recommendations were made as a way of facilitating the knowledge of occupational legislation for workers.
